# Alteration in the sensitivity to crizotinib by Na^+^/H^+^ exchanger regulatory factor 1 is dependent to its subcellular localization in ALK-positive lung cancers

**DOI:** 10.1186/s12885-020-6687-9

**Published:** 2020-03-12

**Authors:** Fenglian Yang, Mu Hu, Siyuan Chang, Jing Huang, Yang Si, Jinghui Wang, Shan Cheng, Wen G. Jiang

**Affiliations:** 1 School of Basic Medical Sciences, Capital Medical University, Beijing, 100069 People’s Republic of China; 2 Beijing Key Laboratory of Cancer & Metastasis Research, Capital Medical University, Beijing, China; 3 Department of Thoracic Surgery, Beijing Xuanwu Hospital, Beijing, 100053 P.R. China; 4 Department of Medical Oncology, Beijing Chest Hospital, Beijing, 101149 P.R. China; 5 Cardiff China Medical Research Collaborative, Cardiff University School of Medicine, Heath Park, Cardiff, CF14 4XN UK

**Keywords:** NHERF1, Crizotinib, ALK, Lung cancer, PDZ protein

## Abstract

**Background:**

Na^+^/H^+^ exchanger regulatory factor 1 (NHERF1) is an important scaffold protein participates in the modulation of a variety of intracellular signal pathways. NHERF1 was able to enhance the effects of chemo-drugs in breast and cervical cancer cells. Anaplastic lymphoma kinase (ALK) fusion mutations are validated molecules targeted therapy in lung cancers, where crizotinib can be used as the specific inhibitor to suppress tumor progression. However, due to the less frequent occurrence of ALK mutations and the complexity for factors to determine drug responses, the genes that could alter crizotinib sensitivity are unclear.

**Methods:**

Both ALK-translocated and ALK-negative lung adenocarcinoma specimens in tissue sections were collected for immunohistochemistry. The possible mechanisms of NHERF1 and its role in the cell sensitivity to crizotinib were investigated using an ALK-positive and crizotinib-sensitive lung adenocarcinoma cell line H3122. Either a NHERF1 overexpression vector or agents for NHERF1 knockdown was used for crizotinib sensitivity measures, in association with cell viability and apoptosis assays.

**Results:**

The expression level of NHERF1 in ALK-translocated NSCLC was significantly higher than that in other lung cancer tissues. NHERF1 expression in ALK positive lung cancer cells was regulated by ALK activities, and was in return able to alter the sensitivity to crizotinib. The function of NHERF1 to influence crizotinib sensitivity was depending on its subcellular distribution in cytosol instead of its nucleus localized form.

**Conclusion:**

Ectopically overexpressed NHERF1 could be a functional protein for consideration to suppress lung cancers. The determination of NHERF1 levels in ALK positive NSCLC tissues might be useful to predict crizotinib resistance, especially by distinguishing cytosolic or nuclear localized NHERF1 for the overexpressed molecules.

## Background

Na^+^/H^+^ exchanger regulatory factor1 (NHERF1) is an important molecule among the four protein family (NHERF1, NHERF2, NHERF3, NHERF4). NHERF1 is generally regarded as a multifunction cellular scaffold protein containing two structural domains of PDZ1 and PDZ2 upstream of an ezrin–radixin–moesin-binding element. NHERF1 is known to interact with a variety of proteins of great importance in human cancers, including platelet-derived growth factor receptor (PDGFR) and phosphatase and tensin homolog (PTEN) [1]. By counterbalancing PI_3_K/Akt oncogenic signals, NHERF1 enhanced cell responses to PDGFR inhibitor [1]. Mutations at NHERF N-terminus within the conserved PDZ domains hampered its interaction with SYK, promoted the progression of breast cancer [2]. NHERF1 binding to the C-terminal of EGFR inhibited its phosphorylation and attenuated downstream signaling [3].

The differential and heterogeneous expression of NHERF1 is related to the progression of several tissues. In polarized colorectal epithelia, NHERF1 distributed at the apical luminal, but disrupted in cancer cases [4]. In transformed cells, cytoplasmic ectopic NHERF1 expression increased cell proliferation and accelerated the development of malignant phenotype [5]. During the early stages of carcinogenesis in colorectal cancers, nuclear NHERF1 was correlated with poor prognosis [4]. In HCC models, NHERF1 and β-catenin colocalized in the nucleus and functioned as a positive regulator of Wnt signaling and contributed to the malignant phenotype [6]. In breast cancer, increased cytoplasmic NHERF1 and decreased membranous localization during ductal carcinoma in situ (DCIS) transformed into invasive and metastatic types [7]. These data implied that NHERF1 could contribute to the progression of cancers based on its expression and cellular distribution.

Lung cancers are the leading types of cancers over the world in both incidence and morbidity, approximately 85% of which are non-small cell lung cancers (NSCLC) [8]. The five-year survival was merely about 15% following optimized chemotherapy procedures [9]. The recent development using targeted therapy has greatly encouraged the screening of novel lung cancer markers for effective drugs or treatment assessments. The anaplastic lymphoma kinase gene (ALK) is a new driving gene of NSCLC following the discovery of EGFR mutations. ALK is a 1620 amino acid transmembrane protein consisting of extracellular ligand binding, transmembrane and intracellular tyrosine kinase domains [10]. In 2011, the US FDA has approved crizotinib to be used for the treatment of ALK-translocated locally advanced or metastatic NSCLC patients [11]. The diagnosis of ALK-translocated lung cancer took place in 3–5% in all NSCLC patients [12]. Although resistance of crizotinib was reported in ALK positive patients subjected to the procedures of targeted therapy, the possible mechanism was poorly understood to date [13]. Comparing to other drugs, genes that influenced the sensitivity of crizotinib were less reported due to the low frequency of ALK mutations in patients.

From previous studies in NSCLC, NHERF1 proteins in the plasma were increased in patients, suggesting NHERF1 might be useful for the evaluating tumor development and progression. A recent study involving 26 cases of NSCLC tumor puncture and 18 cases of matched tissue sections showed that the increase and mislocation of NHERF1 from immunohistochemistry could be used to indicate cancer invasion [14]. It was also reported that NHERF1 elicited pharmacologic effect to enhance drug response of geftinib and imatinib in breast cancers [15]. In cervical cancer cells, NHERF1, as a putative interaction partner, reduced the stability of MRP4 by facilitating its internalization from cell surface to sensitize cisplatin-refractory [16]. These findings prompted us to evaluate the role of NHERF1 in ALK positive NSCLC in connection with the sensitivity to crizotinib treatments.

In the present study, both ALK-translocated and ALK-negative lung adenocarcinoma specimens in tissue sections were collected for immunohistochemistry. An ALK-positive and crizotinib-sensitive lung adenocarcinoma cell line H3122 was used as the laboratory model for experiments. Either a NHERF1 overexpression vector or agents for NHERF1 knockdown was used for crizotinib sensitivity measures, in association with cell viability and apoptosis assays. The results showed that NHERF1 ectopic expression was able to increase the drug sensitivity of crizotinib treatments. Our findings also suggested ALK activity and downstream signals were not only involved to regulated NHERF1 expression, but also affected the subcellular localization. NHERF1 could be a useful referencing marker to evaluate drug resistance, at least the sensitivity of crizotinib in NSCLC cells.

## Methods

### Tissue sample collection and database accession

Lung adenocarcinoma tissues were collected with the approval by the ethics committee from Beijing Xuanwu Hospital. A total of 10 ALK-positive patients were included with the confirmed diagnosis from the pathology department. ALK rearrangements were identified on formalin-fixed, paraffin-embedded (FFPE) tumors using Vysis ALK Break Apart FISH Probe Kit (Abbott Molecular, Abbott Park, IL, USA). Additional 10 ALK-negative lung adenocarcinoma tissues matched for similar clinical stage and grade were randomly selected for comparative studies. All tumor specimens were prepared at the time of surgical resection, and then fixed in 10% neutral buffered formalin for 24 h following an identical SOP. The processed samples were subjected to IHC analyses. For cross-reference, the NHERF1 gene expression data for normal lung or cancerous adenocarcinoma tissues were extracted from GEO dataset (GSE31210), and then used for statistical analyses.

### Chemical reagents and plasmids

Crizotinib was purchased from HARVEY (Beijing, China). The siRNA targeting sequences for NHERF1 was designed as 5′-GCUAU GGCUU CAACC UGCAT T-3′ and 5′-GAAGG AGAAC AGUCG UGAAT T-3′. The synthetic interfering RNA in duplex was transfected into cells seeded in 6-well plates. A scrambled siRNA 5′-UUCUU CGAAC GUGUC ACGUT T-3′ (Gene-Pharma, Suzhou, China) was used in control samples. Heparin was purchased from Bellancom (Beijing, China). The NHERF1 overexpression plasmid fused with a 3 × FLAG tag was prepared as previously described [17].

### Cell culture and transfection

H3122, the human adenocarcinoma ALK-positive NSCLC cell line, harbouring EML4-ALK variant 1 fusion gene was purchased from Kebai Biotechnology Co. Ltd. (Nanjing, China) and maintained in RPMI 1640 (Gibco, Carlsbad, CA, USA) supplemented with 10% fetal bovine serum (FBS) (ExCellBio, Shanghai, China) and 1% P/S antibiotics (Gibco BRC, Paisley, Scotland, UK). The transfection of plasmids or siRNAs were carried out using the Neofect™ DNA transfection reagent (Neofect Biotech, Beijing, China) or Lipofectamine® 2000 transfection reagent (Invitrogen, Paisley, Scotland, UK). The total proteins were extracted at 48 h post-transfection. The protein levels in treated cells following NHERF1 gene overexpression or its siRNA inhibition were determined by western blotting.

### Cytoplasmic and nuclear extracts

Cells were seeded in 10-cm plates with or without treatments for 48 h, and then harvested in 1000 μl of cold phosphate-buffered saline (PBS). Collected cells were centrifuged (4 °C) at 500 g for 2 min, washed twice with cold PBS, and resuspended in 400 μl of cold lysis buffer (10 mM HEPES, 50 mM NaCl, 5 mM EDTA, 1 mM Benzamidine, 0.5% Triton X-100). The lysates were vortexed for 15 s and placed on ice for 20 min. The samples were centrifuged for 1 min (2000 g) at 4 °C and the supernatant were collected as the cytoplasmic fraction. The precipitate was resuspended in 40 μl nuclear lysis buffer. The cytoplasmic or nuclear lysate were placed on ice for 40 min and vortex for 10 min at 15 s with intervals. The final homogenate was centrifuged at 16,000 g for 10 min (4 °C) and the supernatant was used as the nuclear fraction. The prepared samples were stored at − 80 °C for subsequent analyses.

### Western blotting

The treated cells were lysed following a standard protocol. The protein expression was assessed through sodium dodecyl sulfate-polyacrylamide gel electrophoresis (SDS-PAGE) and western blotting analysis. For ALK signaling experiments, confluent cell monolayer of the H3122 cells were serum-free starved 24 h and then exposed to 100 μg/ml heparin for 30 min. The anti-NHERF1 antibody was purchased from BD Transduction Laboratories (BD Biosciences, San Diego, CA, USA). The anti-GAPDH, anti-ERK, anti-p-ERK and anti-Coilin antibodies were purchased from Santa Cruz Biotechnology (Santa Cruz, CA, USA). The anti-AKT, anti-p-AKT (Ser473), anti-ALK, anti-p-ALK (Tyr1604) antibodies were from Cell Signaling Technology (Danvers, MA, USA). The β-action antibody was obtained from ABclonal Biotechnology (Wuhan, Hubei, China). The protein expression levels were semi-quantified by densitometry of the western blot bands using the ImageJ2 software.

### Cell proliferation assay

For each well, a number of 3000 cells were seeded in 96-well plates and continuously cultured for 0, 1, 2, 3 and 4 days. The cell growth was assayed by adding 100 μl of cell counting kit (CCK8) (Dojindo, Kamimashiki-gun, Kumamoto, Japan) solution at 1:10 dilution in culture medium for staining at 37 °C for 1 h. The plates were scanned at absorbance wavelength of 450 nm using a spectrophotometer (BioTek, Winooski, VT, USA) for detection. Each data point was measured in six duplicates. The experiments were repeated for 3 times independently.

### Apoptosis assay

Cells were seeded in 6-well plates at the density of 1 × 10^5^ cells/well. The treatments with crizotinib at 10 μM were maintained for 24 h. In the control group, the cells were treated vehicle (DMSO) only for dissolving the drugs. The cell apoptosis was assessed using a MUSE™ Annexin V Dead Cell Kit (EMD Millipore Corporation, Hayward, CA, USA) following the manufacturer’s protocol.

### Immunofluorescence

Cells were plated at a density of 1.0 × 10^5^ per well into 6-well plates pre-placed with a glass coverslip. Following transfection, the cells on coverslips were treated with/without heparin and then washed three times with cold PBS. After fixed with 4% paraformaldehyde at room temperature for 30 min, the cells were permeabilized and blocked with a buffer containing 0.3% Triton X-100 and 5% bovine serum albumin for 30 min. To determine the subcellular localization of NHERF1, cells were probed with monoclonal mouse anti-human NHERF1 primary antibody (1:100) (BD Transduction Laboratories) and anti-Flag primary antibody (1:200) (Sigma-Aldrich, St. Louis, MO, USA). Following extensive wash for removing the primary antibodies, the cells were incubated with an Alexa Fluor® 488 donkey anti-mouse IgG (Life Technologies, MA, USA) at 1:200 dilution in the dark for 1 h. Prior to the examination under a fluorescent microscope, cell nuclei were stained with Hoechst 33258 (Sigma-Aldrich). Images were acquired with a confocal system (Leica Microsystems LAS AF-TCS SP5. Wetzlar, Germany). Control samples without adding primary or secondary antibodies were prepared for evaluating the non-specific staining in this study.

### Immunohistochemistry (IHC)

The 5 μm sections of collected lung tissues were deparaffinized, rehydrated, rinsed with distilled water, and washed with Tris-buffered saline (TBS). Automated IHC staining was carried out using a Ventana BenchMark GX instrument (Ventana Medical Systems, Inc., Tucson, AZ, USA). NHERF1 was probed with an anti-NHERF1 antibody (1:100, Abcam, Cambridge, MA, USA). Image Pro Plus 6.0 (Media Cybernetics, Inc., Rockville, MD, USA) was used to calculate the intensity of NHERF1 staining. Randomly selected 10 microscopic fields were imaged for each sample subjected to computer-assisted imaging analyses. The results were presented as the mean ± standard deviation (SD). The determination of immunohistochemical staining intensity and positive areas for NHERF1 in IOD (integral optical density) measures were performed by two independent staffs who were mutually blinded to the sample grouping information.

### Statistical analyses

The quantitative data were presented as mean ± SD and subjected to Student t-test. Statistical significance was called at *p* < 0.05. The charts were generated by Prism V5.0 (GraphPad Software, Inc., La Jolla, CA, USA).

## Results

### NHERF1 expression was increased in ALK positive lung adenocarcinoma tissues

**Fig. 1:**
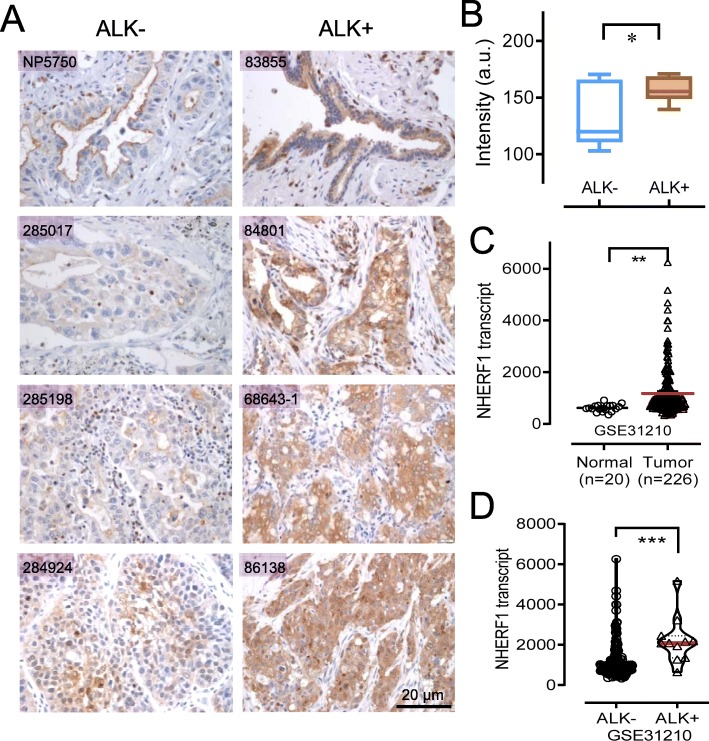
Upregulated levels of NHERF1 expression were observed in ALK positive lung adenocarcinoma tissues. **a** Detection of NHERF1 protein by immunohistochemistry in tissue sections from ALK-translocated lung adenocarcinoma patients and compared to matched controls. **b** Quantification of (**a**) when *n* = 10 in both groups. **c** Relative NHERF1 mRNA levels in lung adenocarcinoma tissues (*n* = 226) from Set GSE31210 in the GEO database and the normal lung tissues (*n* = 20). **d** Stratified NHERF1 mRNA levels in ALK-translocated lung adenocarcinoma tissues (*n* = 11) and ALK negative samples (*n* = 215). *p < 0.05, **p < 0.01;***p < 0.001

Using the ALK-positive and ALK-negative lung adenocarcinoma specimens collected from Beijing Xuanwu Hospital, we analysed the expression of NHERF1 by immunohistochemistry on tissue sections. We found that the NHERF1 staining was much more intensive in samples of ALK-translocated tissues, compared to that in ALK-negative lung adenocarcinoma tissues (Fig. 1a). The subsequent statistics with the IHC semi-quantification indicated that the difference was significant (*p* < 0.05) (Fig. 1b). We also evaluated the NHERF1 mRNA expression using the data extracted from GEO dataset (GSE31210), where the mRNA expression pattern in 246 tissue specimens was documented. Relative NHERF1 mRNA in lung adenocarcinoma was over the normal tissues (*p* < 0.01) (Fig. 1c). Increased level of NHERF1 was again found to be more significant for ALK-translocated tissues and compared to those in the tissues of ALK negative samples (*p* < 0.001) (Fig. 1d). Not only we supplied additional data of the NHERF1 expression at the protein levels, we argue that besides the high relative NHERF1 mRNA in lung adenocarcinoma over the normal tissues, whether the elevated level of NHERF1 is associated with the abnormal activation of ALK or related signaling needs to be investigated.

### The upregulation of NHERF1 expression was dependent to the activation of ALK signaling

**Fig. 2:**
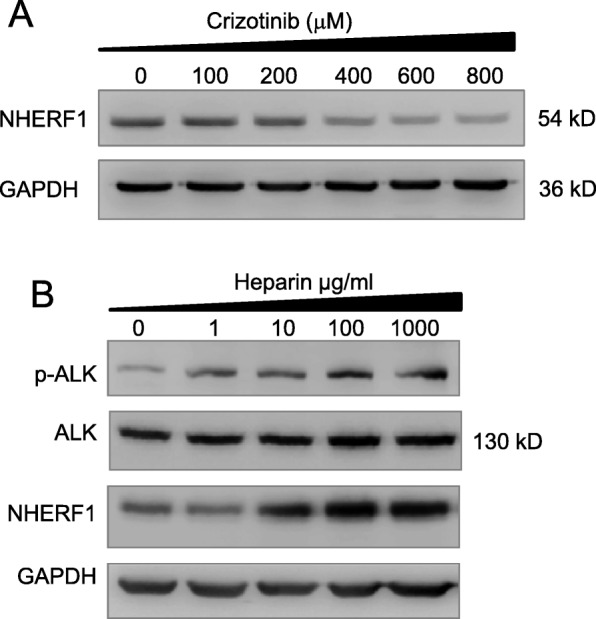
Effect of ALK signaling on NHERF1 expression (**a**) The expression of NHERF1 during the time course of crizotinib treatment for 7 days in H3122 cells (Full-length blots/gels are presented in Suppl Figure 2). **b** A dose dependent autophosphorylation of ALK through heparin stimulation increased NHERF1 expression in H3122 cells. (Full-length blots/gels are presented in Suppl Figure 2)

To address whether NHERF1 expression could be a consequence upon the activation of ALK signaling, we conducted laboratory experiment in H3122 cells, which is known as a commonly used ALK-positive and crizotinib-sensitive lung adenocarcinoma cell line. As crizotinib is the specific inhibitor of ALK, with which the treatment in H3122 cells significantly reduced the level of NHERF1 for its expression (Fig. 2a). Heparin is a potent activating ligand of ALK for its receptor tyrosine kinase activity. When cells treated with heparin, robust autophosphorylation of ALK can be observed. In heparin treated H3122 cells, the endogenous expression of NHERF1 was significantly increased and was in a dose dependent fashion (Fig. 2b). These data suggested the elevated NHERF1 expression levels could very well be a subsequent response following the activation of ALK and downstream signaling in ALK-translocated lung adenocarcinomas.

### Knockdown of NHERF1 expression attenuated the sensitivity of H3122 cells to crizotinib

**Fig. 3:**
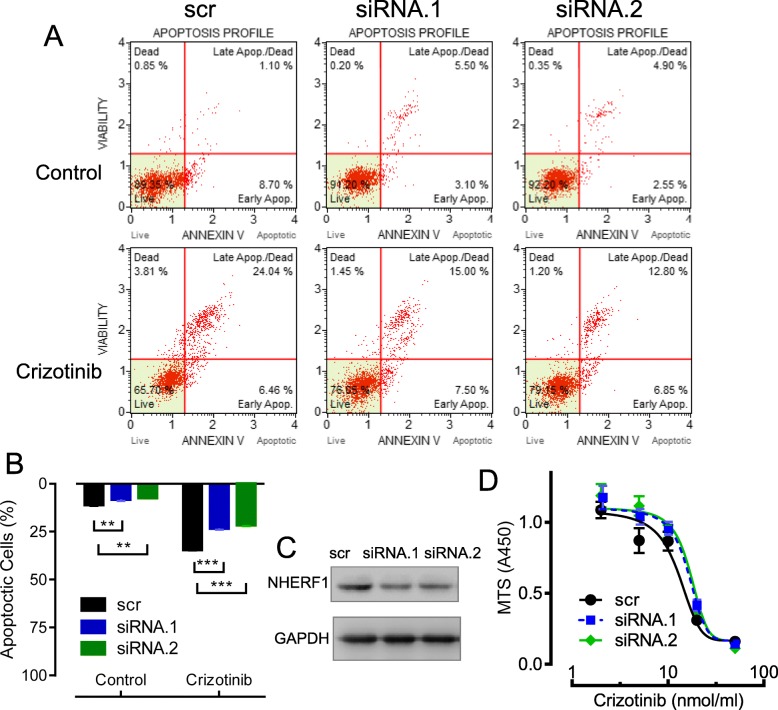
Effects of NHERF1 knockdown on the sensitivity of H3122 cells to crizotinib. **a** Rate of cell apoptosis by flow cytometry with 10 μM crizotinib treatments. **b** Quantification and statistics from (**a**) and repeated experiments (*n* = 3). **c** The knockdown of NHERF1 at protein levels as determined by western blotting (Full-length blots/gels are presented in Suppl Figure 3). **d** The cell survival from MTS assays in H3122 and NHERF1 knockdown cells in the presence of crizotinib (0, 2, 5, 10, 20 and 50 μM). **p < 0.01, ***p < 0.001

From previous reports in breast cancer and cervical cancer cells, NHERF1 was shown to be able to influence the sensitivity of a variety of anti-cancer drugs [18, 19]. We started to wonder if such pharmacologic functions of NHERF1 can also be observed in lung cancers, especially in ALK positive lung cancer cells. We used the transfection of interfering siRNAs and prepared NHERF1 knockdown models in H3122 background. The expression level of NHERF1 was considerably decreased in transfected cells as shown by western blotting, compared with scramble control cells (Fig. 3c). The sensitivity to crizotinib were assayed through the measures in the cell viability and rate of apoptosis in transfected H3122 cells. The cell apoptosis was observed to be significantly inhibited following 10 μM crizotinib treatments, as shown in Fig. 3a,b by flow cytometry (from 35 to 22% or 19%). By knockdown of NHERF1 expression in H3122 cells, Increased cell survival was observed from the crizotinib dose administration curve (Fig. 3d). These results indicated that the knockdown of NHERF1 could reduce the sensitivity to crizotinib in ALK-positive cells.

### Knockdown of NHERF1 promoted cell growth and enhanced ALK activation

**Fig. 4:**
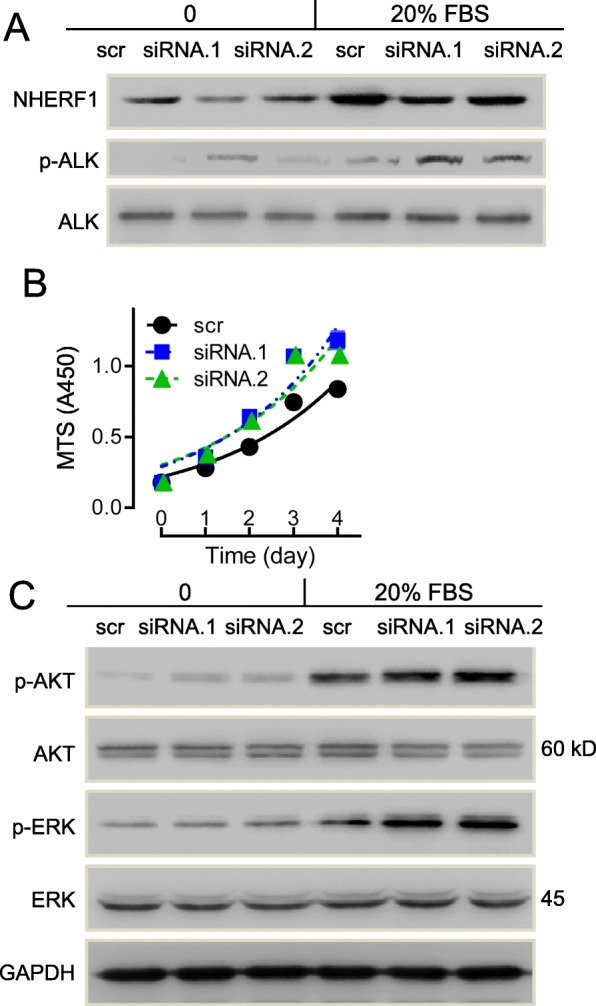
Effects of NHERF1 Knockdown on cell viability and related signals. **a** Knockdown expression of NHERF1 significantly promoted FBS-induced ALK activation in H3122 cells (Full-length blots/gels are presented in Suppl Figure 4). **b** MTS assays for cell growth in H3122 and NHERF1 knockdown cells (*n* = 6). **c** Knockdown expression of NHERF1 on the expression and phosphorylation of AKT and ERK. (Full-length blots/gels are presented in Suppl Figure 4)

To further examine whether NHERF1 knockdown in ALK positive cells indeed alters the ALK kinase activity thus to alter crizotinib sensitivity, we analyzed the phosphorylation of ALK and related signal molecules. In NHERF1 knockdown H3122 cells, the levels of phosphorylated ALK were significantly increased (Fig. 4a) following 15 min stimulation with 20% FBS, which is a known treatment for activating ALK. In addition, the phosphorylation of AKT and ERK were also increased (Fig. 4c). As both ERK and AKT were possible downstream proteins of ALK to regulate cell growth and proliferation, we evaluated the effect of NHERF1 knockdown on growth. As shown in Fig. 4b, the increase in CCK8 assays of 125 and 150% was observed following the transfection of two pairs of siRNAs, respectively. These data suggested that reduction in NHERF1 expression led to the increased of cell growth in H3122 cells, which involved the increased phosphorylation of ALK and subsequent enhancement in downstream signal activation of AKT and ERK. This at least partially explained previously discovered crizotinib sensitivity loss after RNAi of NHERF1, where the accompanied increase in ALK activation is expected to require increased drug dose to achieve the same level of induction for apoptosis.

### Overexpression of NHERF1 inhibited the activation of ALK signal and suppressed cell growth

**Fig. 5:**
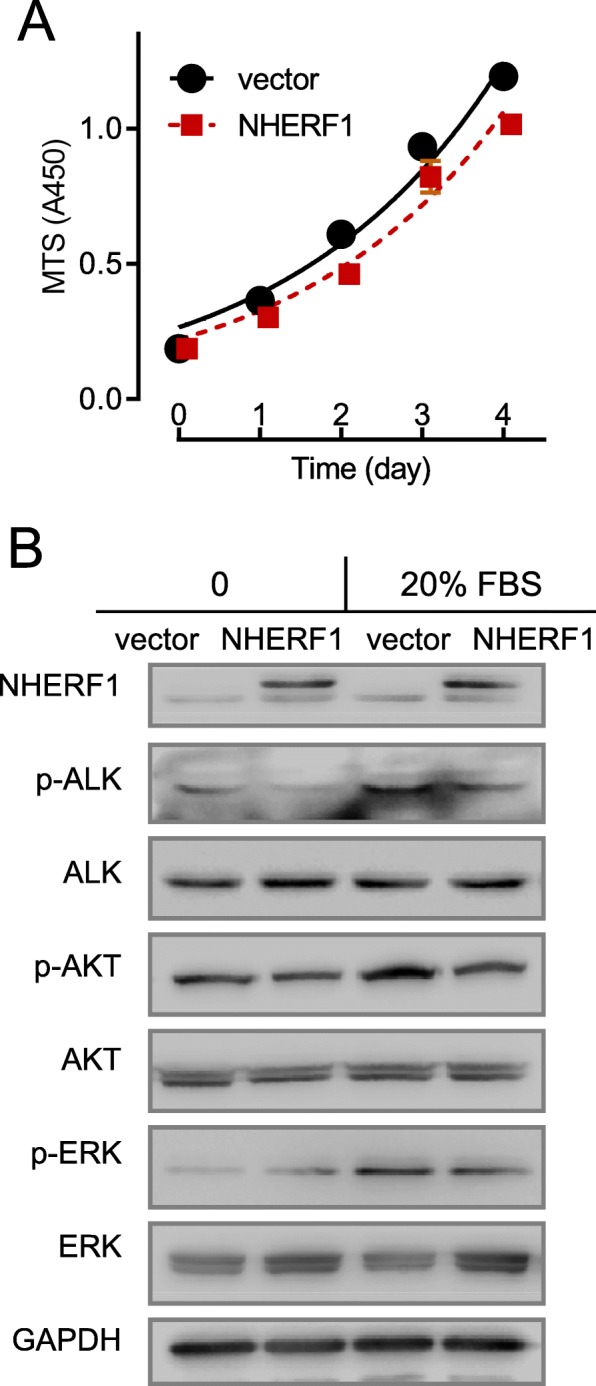
Effects of NHERF1 overexpression on cell viability and related signals. **a** MTS assays for cell growth in H3122 and NHERF1 overexpressed cells (*n* = 6). **b** Over-expression of NHERF1 significantly inhibited FBS-induced ALK, AKT and ERK activation (Full-length blots/gels are presented in Suppl Figure 5)

We next explored on whether NHERF1 overexpression in H3122 cells will give a reversed phenotype comparing to the knockdown situation. A plasmid carrying an expression cassette of NHERF1 was transfected into H3122 cells, and the protein levels were assessed by western blotting (Fig. 5b). A mild reduction of cell growth to 80% was observed in NHERF1 transfected cells comparing to the vector control (Fig. 5a). However, the suppression on FBS-induced ALK phosphorylation and associated AKT and ERK activation appeared to be more substantial under NHERF1 overexpression (Fig. 5b). The results suggested that NHERF1 overexpression decreased activation of ALK and associated ERK or AKT signaling. It is also implied that the resulted inhibition on apoptosis seemed to be more significant than that on cell growth.

### NHERF1 overexpression sensitized H3122 cells to crizotinib treatments

**Fig. 6:**
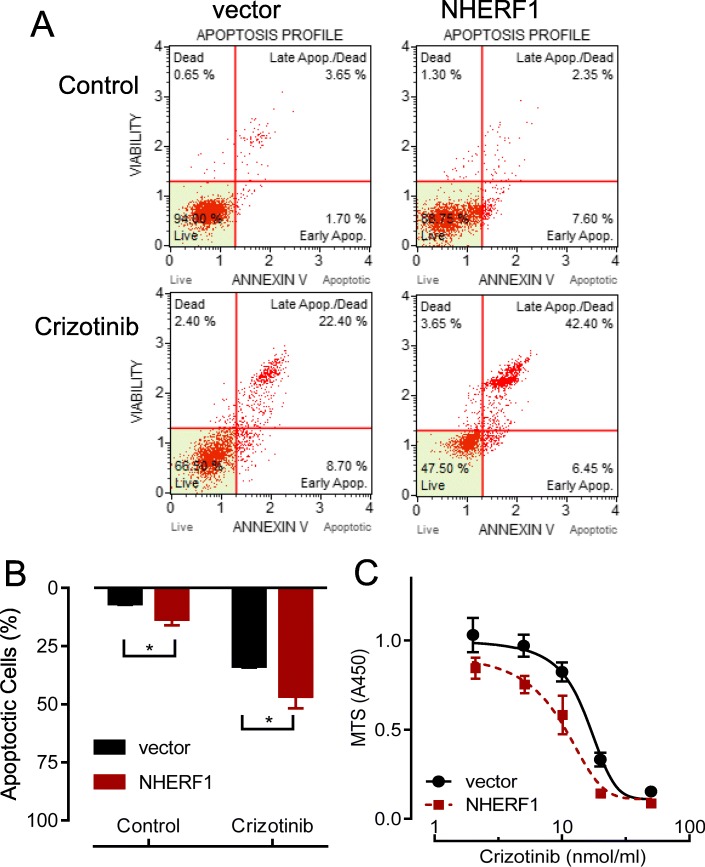
Effects of NHERF1 overexpression on the sensitivity of H3122 cells to crizotinib. **a** Rate of cell apoptosis by flow cytometry with 10 μM crizotinib treatments. **b** Quantification and statistics from (**a**) and repeated experiments (*n* = 3). **c** The cell survival from MTS assays in H3122 and NHERF1 overexpressed cells in the presence of crizotinib (0, 2, 5, 10, 20 and 50 μM). *p < 0.05

Indeed from the crizotinib sensitivity assays, the apoptosis of H3122 cells increased to 12% in NHERF1 overexpressed cells from a basal level of 7% in the vector controls. When cells exposed to 10 μM crizotinib for 24 h, a 35% apoptosis rate increased to 47% with ectopic overexpression of NHERF1 in H3122 cells (Fig. 6a,b). Besides, overexpression of NHERF1 significantly reduced the cell survival in the presence of crizotinib as compared to the empty vector-transfected control cells (Fig. 6c). These results verified our findings from previous experiments demonstrated the importance of NHERF1 in ALK-translocated lung adenocarcinoma cancer cells.

### Ectopically expressed NHERF1 differed from the endogenous proteins in subcellular localization in cancer cells and exerted more potent inhibition on cell growth and drug sensitivity

**Fig. 7:**
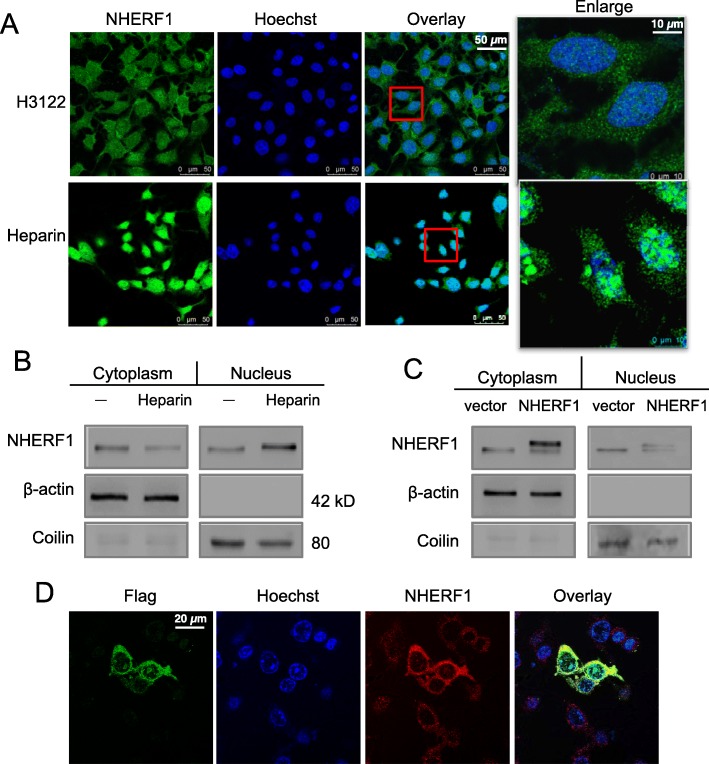
Subcellular localization of endogenous, ALK-induced or ectopic overexpressed NHERF1 proteins in H3122 cells. **a** Immunofluorescent microscopy in native H3122 cells (upper) or cells treated with Heparin (100 μg/ml) for 30 min (lower). **b** Western blot analyses for cytoplasm or nuclear distribution of NHERF1 proteins, where Coilin was used as a nuclear maker (Full-length blots/gels are presented in Suppl Figure 6). **c** Western blot of NHERF1 in cells transfected with a Flag-NHERF1 plasmid for ectopic overexpression (Full-length blots/gels are presented in Suppl Figure 6). **d** Confocal microscopy for distinguishing ectopic NHERF1 vs. the endogenous protein

Although NHERF1 is generally consensed as a gene with tumor suppressor functions, it is not quite understood why it often upregulated in malignant cancer cells. Recent studies showed that the cellular distribution of NHERF1 was significantly different in cancers from normal tissues of same origins, where NHERF1 protein might shifted from the plasma membrane to the cytoplasm or nucleus [20]. In our experiments, immunofluorescence was used to examine the subcellular localization of NHERF1 in H3122 cells. From the representative images, endogenous NHERF1 could be identified at membrane structures, as well as in the cytoplasm or nuclei (Fig. 7a Upper). When incubated cells in 100 μg/ml heparin, the increased expression of NHERF1 proteins was significant as predominantly found in the nuclei of H3122 cells (Fig. 7a Lower). Nevertheless, the ectopic overexpression of NHERF1 through plasmid transfection resulted in the protein to be distributed with its natural existence in the membrane or cytoplasm (Fig. 7c, d). From the western blot individually probing NHERF1 in the cytoplasmic and nuclear forms, the found pattern could be confirmed (Fig. 7b, c). We postulated that the ALK activation induced NHERF1 tended to be selectively transported into the nuclei, and such mislocalized NHERF1 proteins were likely abolished the normal functions to suppress the growth and survival of cancer cells.

## Discussion

The upregulation of NHERF1, as a suggestive poor prognosis indication, was earlier discovered in breast cancers for its association with tumor malignancy and metastatic progression [21]. Latest studies attempted to discriminate the diverged functions of NHERF1 with different subcellular distribution. Cytoplasmic NHERF1 was detected higher in primary cancer than in adjacent normal mucosa, and implicated nodal and distant metastases or lymphovascular invasion (LVI) [7]. Nuclear NHERF1 was identified in colorectal cancer of early stages and was believed to participate the onset of the malignant phenotype during carcinogenesis leading to poor prognosis [22]. In lung cancers, NHERF1 protein was increased in both plasma and tissues from NSCLC patients. NHERF1 interaction partners can be either cytoplasmic or nuclear factors, regulated by a wide range of signals in breast cancer or colorectal cancers, such as VEGFR and HIF-1ɑ, some of which closely associated with lymphatic metastasis [17, 23]. Besides HIF-1ɑ, NF-κB was another important nuclear transcription factor demonstrated to interact with NHERF1 dependent to the activation of Ras/MEK/ERK or PI3K/Akt signaling [24]. In primary macrophages and vascular smooth muscle cells, NF-κB in return was shown to promote the expression of NHERF1. From this study, a high level of NHERF1 expression was found in lung cancer tissues, especially in patients diagnosed with ALK-translocation (Fig. 1). ALK was a known activator of AKT and ERK signaling pathways upstream of NF-κB or HIF-1ɑ mediated transcription of the regulated genes, therefore it was worthwhile to exploit whether the activation of ALK signal was sufficient or responsible to modulate NHERF1 expression. As predicted, we found that ALK activation induced by heparin in coordination with ERK and AKT phosphorylation upregulated the transcription and expression of NHERF1 (Fig. 2b). The result immediately suggested that NHERF1 might influence the sensitivity to crizotinib in ALK targeted therapies, as implied from Fig. 2a.

NHERF-interacting proteins, comprising transporters, membrane receptors, junction proteins or signaling molecules, have been identified with a function to influence the cell survival following treatments of chemo-drugs [19]. It is no surprise for NHERF1 to alter the sensitivity or resistance to cancer drugs. In fact, NHERF1 has also been suggested to connect with tumor sensitivity to drugs targeting EGFR, MRP4 or MRP2 [18]. The knockdown of NHERF1 expression reduced crizotinib sensitivity in ALK-positive H3122 cells via its inhibition on cell apoptosis (Fig. 3a,d) and promotion of cell growth (Fig. 4a). Further results from NHERF1-overexpressed H3122 cells (Figs. 5 and 6) were able to verify the role of NHERF1 on crizotinib sensitivity, except for the effects on cell growth was weak. These results were consistent with the ectopic overexpression of NHERF1 significantly reduced the heparin-induced cell growth in H3122 cells (suppl fig. 1). NHERF1 is a scaffold protein, which can form complex network through its two PDZ domains to regulate many cellular signal pathways [16]. A majority of the molecular interactions involving the binding of NHERF1 is dependent to protein phosphorylation [1]. The dentification for major NHERF1 interaction protein with its chemical modification status will be particularly helpful to understand the exact mechanism explaining the effect of NHERF protein on drug sensitivities. To the best of our knowledge, there is no evidence indicating a direct interaction between NHERF and ALK, the connection of NHERF1 to ALK activation was not previously reported and needed to be further studied.

NHERF1 was suggested to play an essential role in cancer initiation, development, progression and metastasis [6]. An important finding from this study was the differential effect of NHERF1 functions linked to its localization. Ectopic expression of NHERF1 preferred a cytoplasm distribution and led to strong inhibition of cell growth (Figs. 5 and 7). Thus, it remained to be considered as a potential candidate for manipulation to treat cancers as previously recognized. The nuclear NHERF1 played a role on promoting tumor progression through the interaction with YAP and increased YAP translocation into the nucleus for transcription activation [25]. In a few reports, it was also implied that NHERF1 lost its tumor suppressive function once mislocated from the cytoplasm into the nucleus, or even worse acted as a pro-oncogene product. We found that the translocation of NHERF1 happened to heparin-induced endogenous NHERF1 expression in ALK positive cancer cells (Fig. 7). The results partially explained why malignant cancer cells not only sustained high NHERF1 expression but also might benefit from the outcome for proliferation preference. Continuous explore about the complex mechanisms of NHERF1 regulating functions in cancers will supply more evidence to address the remaining questions.

Crizotinib is a first-generation ALK-TKI applied to NSCLC subtypes. Resistance to crizotinib can occur via secondary mutations or amplification in the ALK gene. Cancer cells may adopt various strategies, such as amplified KIT signaling, increased EGFR phosphorylation, KRAS mutations oncogene, or increased NRG1 for activating HER2/3 kinases to survive crizotinib treatments [26]. Epithelial-mesenchymal transition (EMT) was suggested to be a critical process to confer resistance to crizotinib in NSCLC. NHERF1 suppressed EGFR phosphorylation and inhibited EGF-induced proliferation/migration in breast cancer cells [27]. Previous studies showed that NHERF1 suppressed lung cancer cell migration by attenuating EMT process. Loss of the interaction between NHERF1 and EGFR induced EMT phenotypic features in biliary cancer cells [28]. We found crizotinib reduced the expression of NHERF1 (Fig. 2a), implying that the inhibition of NHERF1 on EGFR phosphorylation was compromised. NHERF1 was discovered as putative interaction partners of MRP4 [16] in cervical cancers [19]. It implied that monitoring changes in NHERF1 levels could be used to predict loss of crizotinib sensitivity during drug treatments, as the inhibitory function of NHERF1 to EMT contributed to crizotinib drug resistance.

The role of NHERF1 in drug resistance in lung cancers deserves more investigation for clues to understand the underlining mechanism. By characterization the effects of NHERF1 with its regulation on tumor cell responses to crizotinib, we have demonstrated a dynamic interplay between NHERF1 and ALK signaling in lung adenocarcinoma cells. Upregulation of NHERF1 in lung adenocarcinoma needs to be scrutinized during the administration of ALK-targeting drugs. The reduction in NHERF1 expression in cancer cells treated with crizotinib is likely to suggest acceleration of tumor progression, as well as the resistance to drugs. The expression and subcellular distribution of NHERF1 might be a potential prognostic factor to predict the benefit in patients subjected to ALK targeted therapies.

## Conclusion

The present study not only reemphasized NHERF1 as a potential marker to evaluate the malignancy and progression in ALK-translocated non-small cell lung cancers, similar to what has been reported in other cancer types, but also further demonstrated the increased NHERF1 expression was regulated by ALK activation. The sensitivity to ALK-targeted drug crizotinib was related ERK or AKT signals to influence cell growth and apoptosis. The ALK-induced NHERF1 expression appeared to have distinct cellular localization from ectopic overexpressed forms, which contributed differently to cell survival, growth and drug sensitivity. Our findings provided a new perspective for understanding the underlying mechanisms for ALK-translocated lung adenocarcinoma to survive from crizotinib treatments.

## Supplementary information

**Additional file 1.**

**Additional file 2.**

## Data Availability

Public online or upon request.

## Abbreviations

ALK: Anaplastic lymphoma kinase
CCK8: Cell counting kit
DCIS: Ductal carcinoma in situ
EMT: Epithelial-mesenchymal transition
FBS: Fetal bovine serum
FFPE: Formalin-fixed, paraffin-embedded
IHC: Immunohistochemistry
IOD: Integral optical density
LVI: Lymphovascular invasion
NHERF1: Na^+^/H^+^ exchanger regulatory factor 1
NSCLC: Non-small cell lung cancers
PBS: Phosphate-buffered saline
PDGFR: Platelet-derived growth factor receptor
PTEN: Phosphatase and tensin homolog
SD: Standard deviation
SDS-PAGE: Sodium dodecyl sulfate-polyacrylamide gel electrophoresis
TBS: Tris-buffered saline
