# Supplementary material for: Alteration in the sensitivity to crizotinib by Na+/H+ exchanger regulatory factor 1 is dependent to its subcellular localization in ALK-positive lung cancers
Source: BMC Cancer. 2020 Mar 12;20:202. doi: 10.1186/s12885-020-6687-9 (PMC7068933; doi:10.1186/s12885-020-6687-9)
Supplement: Supplementary file 1 — Additional file 1. [file 12885_2020_6687_MOESM1_ESM.pdf]

Suppl figure 1 for publication

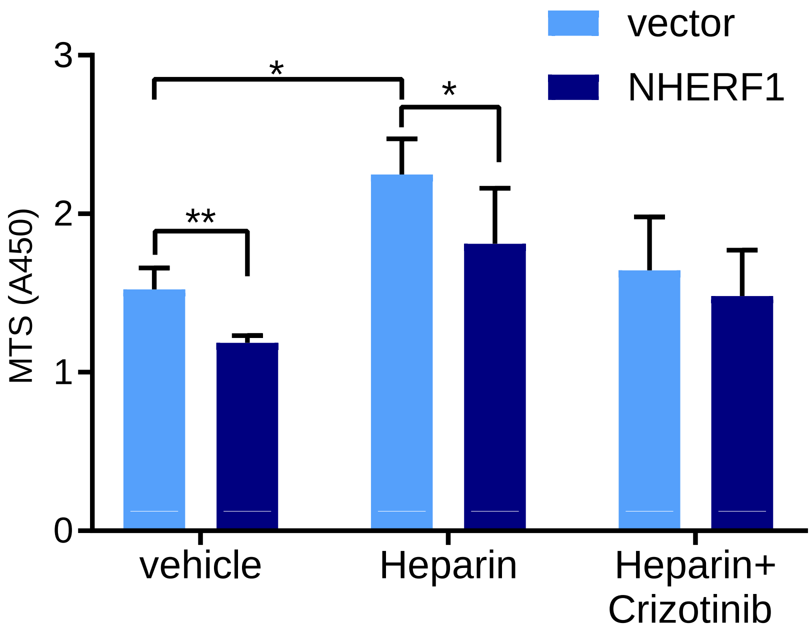

Suppl figure 1. The cell viability from MTS assays in H3122 and NHERF1 overexpressed cells in the presence of heparin (1 µg/ml), or crizotinib (1 µM), or both for 72 h. *p<0.05, **p<0.01.
