# Supplementary material for: Alteration in the sensitivity to crizotinib by Na+/H+ exchanger regulatory factor 1 is dependent to its subcellular localization in ALK-positive lung cancers
Source: BMC Cancer. 2020 Mar 12;20:202. doi: 10.1186/s12885-020-6687-9 (PMC7068933; doi:10.1186/s12885-020-6687-9)
Supplement: Supplementary file 2 — Additional file 2. [file 12885_2020_6687_MOESM2_ESM.pdf]

Suppl figure 2

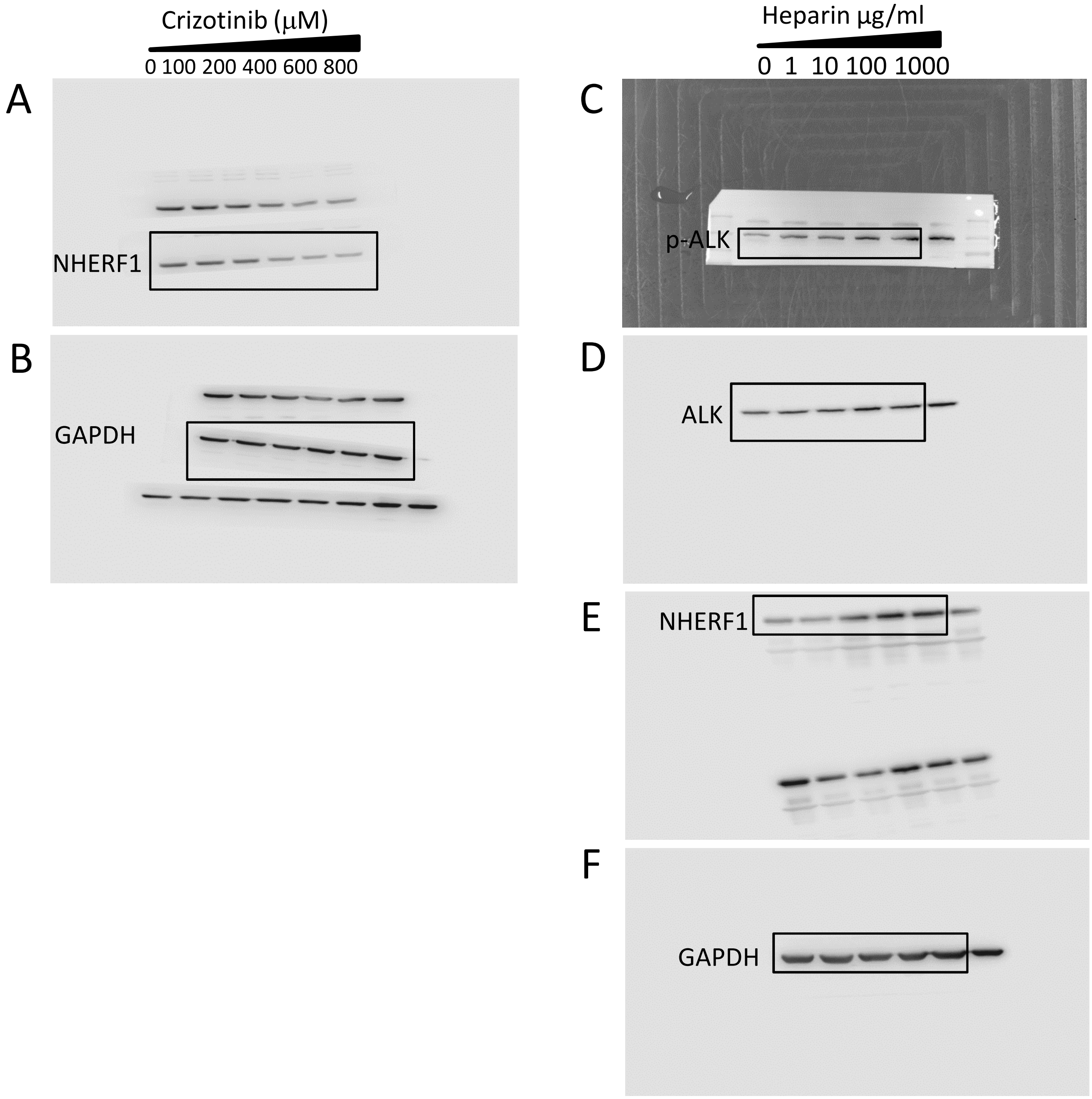

Suppl figure 2 (original images to Figure 2). (A) Original image to Figure 2A-NHERF1. (B) Original image to Figure 2A-GAPDH. (C) Original image to Figure 2B-p-ALK. (D) Original image to Figure 2B-ALK. (E) Original image to Figure 2B-NHERF1. (F) Original image to Figure 2B-GAPDH.

Suppl figure 3

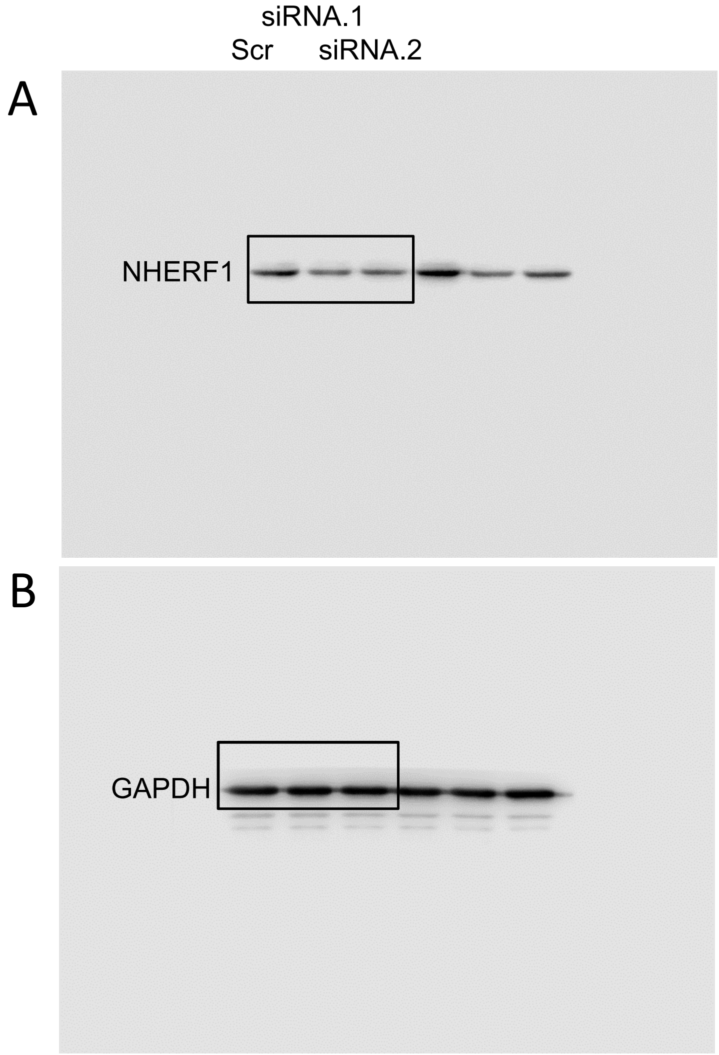

Suppl figure 3 (original images to Figure 3). (A) Original image to Figure 3C-NHERF1. (B) Original image to Figure 3C-GAPDH.

Suppl figure 4

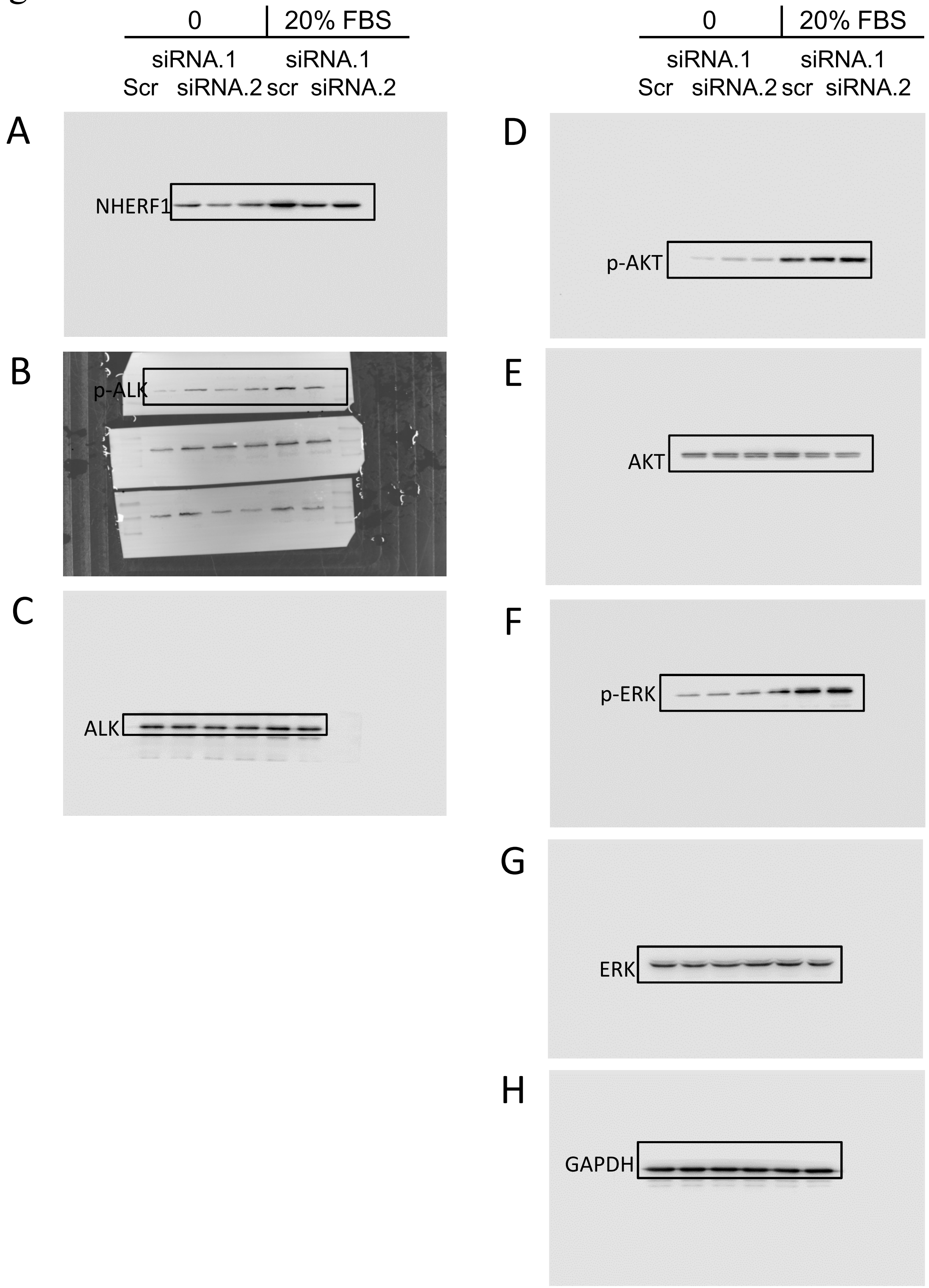

Suppl figure 4 (original images to Figure 4). (A) Original image to Figure 4A-NHERF1. (B) Original image to Figure 4A-p-ALK. (C) Original image to Figure 4A-ALK. (D) Original image to Figure 4C-p-AKT. (E) Original image to Figure 4C-AKT. (F) Original image to Figure 4C-p-ERK. (G) Original image to Figure 4C-ERK. (H) Original image to Figure 4C-GAPDH.

Suppl figure 5

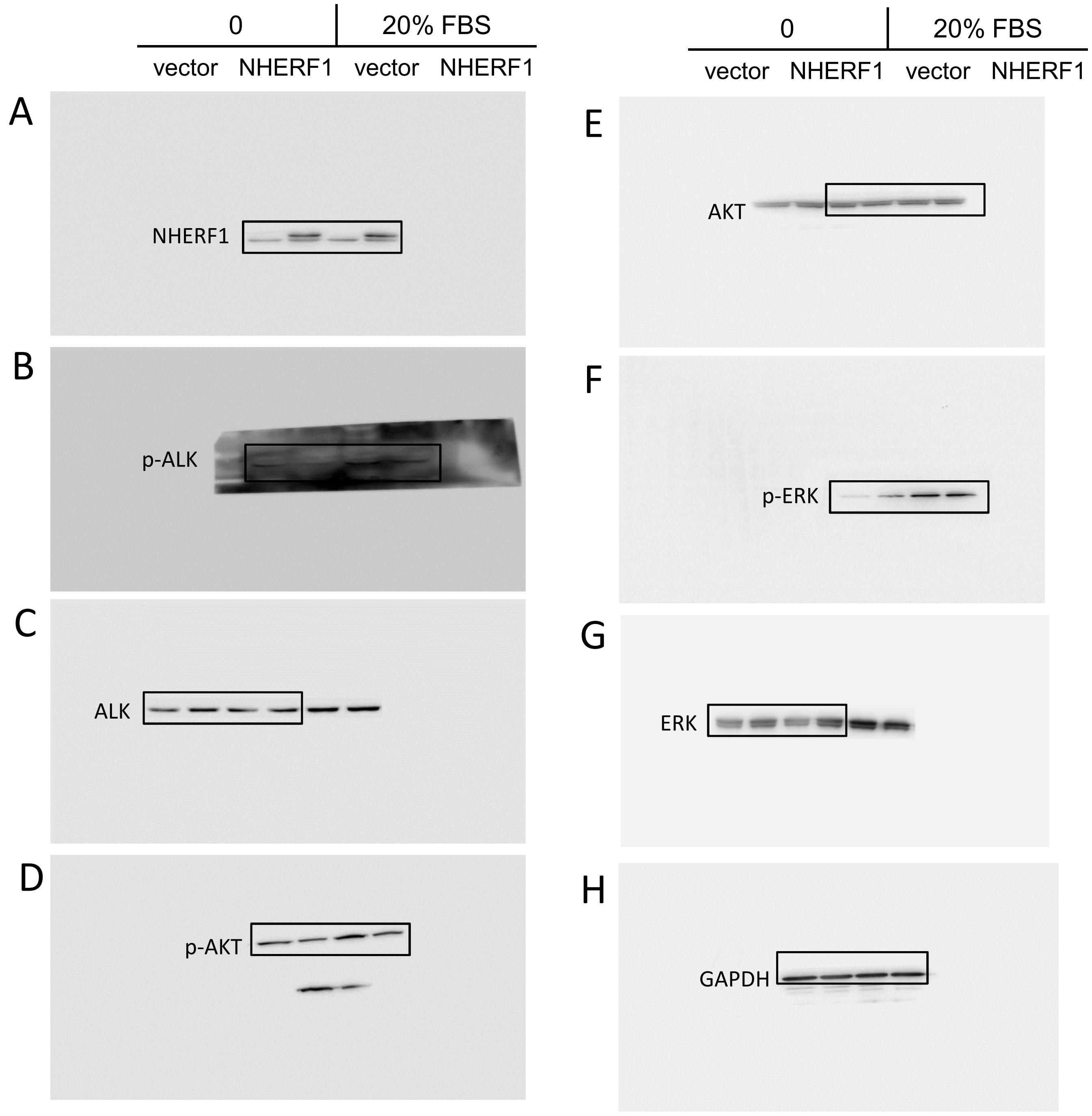

Suppl figure 5 (original images to Figure 5). (A) Original image to Figure 5B-NHERF1. (B) Original image to Figure 5B-p-ALK. (C) Original image to Figure 5B-ALK. (D) Original image to Figure 5B-p-AKT. (E) Original image to Figure 5B-AKT. (F) Original image to Figure 5B-p-ERK. (G) Original image to Figure 5B-ERK. (H) Original image to Figure 5B-GAPDH.

Suppl figure 6

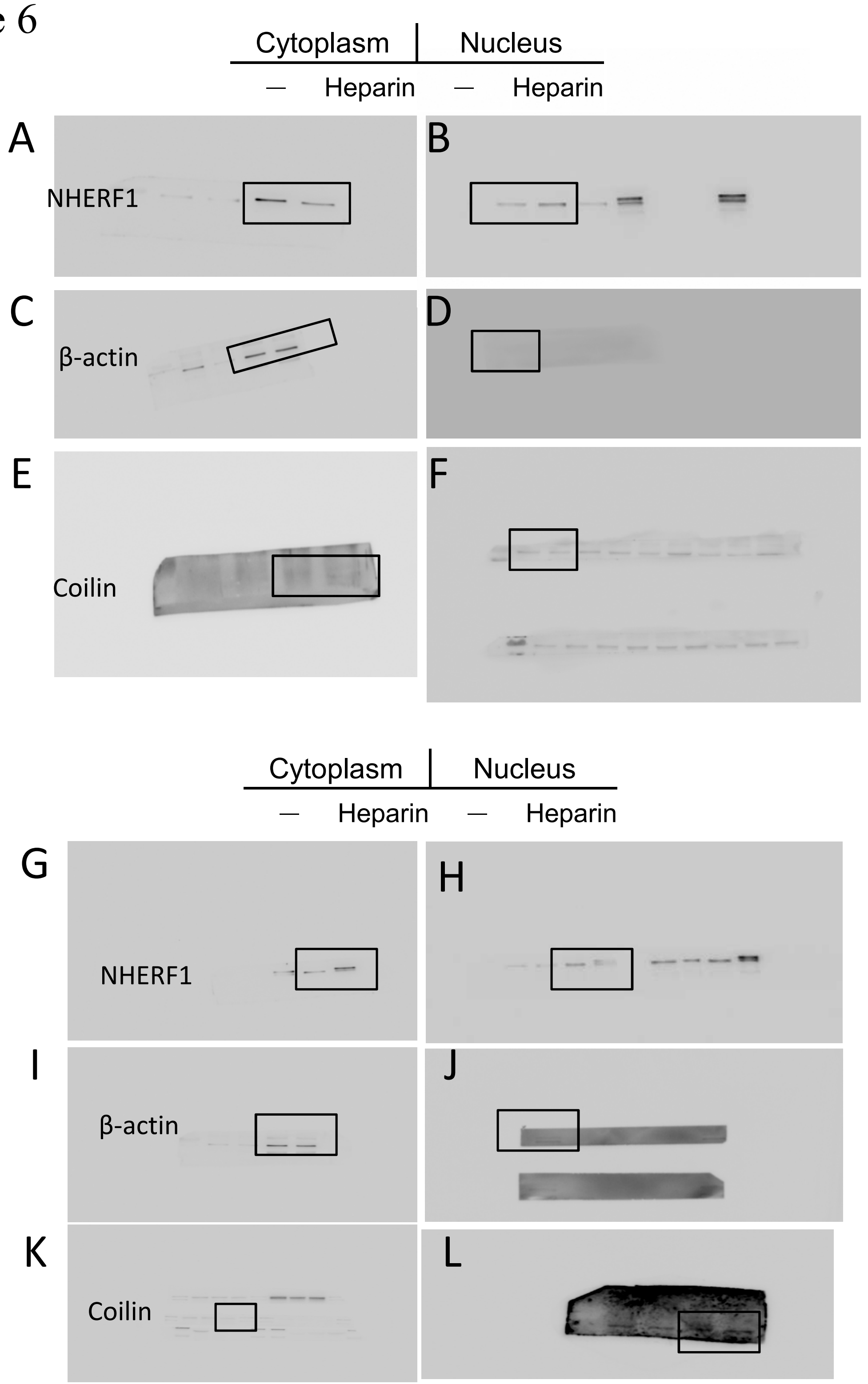

Suppl figure 6 (original images to Figure 7). (A) Original image to Figure 7B-Cyto-NHERF1. (B) Original image to Figure 7B-Nu-NHERF1. (C) Original image to Figure 7B-Cyto-actin. (D) Original image to Figure 7B-Nu-actin. (E) Original image to Figure 7B-Cyto-Coilin. (F) Original image to Figure 7B-Nu-Coilin. (G) Original image to Figure 7C-Cyto-NHERF1. (H) Original image to Figure 7C-Nu-NHERF1. (I) Original image to Figure 7C-Cyto-actin. (J) Original image to Figure 7C-Nu-actin. (K) Original image to Figure 7C-Cyto-Coilin. (L) Original image to Figure 7C-Nu-Coilin.
